# Complete Sequence and Analysis of the Mitochondrial Genome of *Hemiselmis andersenii *CCMP644 (Cryptophyceae)

**DOI:** 10.1186/1471-2164-9-215

**Published:** 2008-05-12

**Authors:** Eunsoo Kim, Christopher E Lane, Bruce A Curtis, Catherine Kozera, Sharen Bowman, John M Archibald

**Affiliations:** 1 Canadian Institute for Advanced Research, Integrated Microbial Biodiversity Program, Department of Biochemistry and Molecular Biology, Dalhousie University, Halifax, Nova Scotia, Canada; 2 The Atlantic Genome Centre, Halifax, Nova Scotia, Canada; 3 Department of Process Engineering and Applied Science, Dalhousie University, Halifax, Nova Scotia, Canada

## Abstract

**Background:**

Cryptophytes are an enigmatic group of unicellular eukaryotes with plastids derived by secondary (i.e., eukaryote-eukaryote) endosymbiosis. Cryptophytes are unusual in that they possess four genomes–a host cell-derived nuclear and mitochondrial genome and an endosymbiont-derived plastid and 'nucleomorph' genome. The evolutionary origins of the host and endosymbiont components of cryptophyte algae are at present poorly understood. Thus far, a single complete mitochondrial genome sequence has been determined for the cryptophyte *Rhodomonas salina*. Here, the second complete mitochondrial genome of the cryptophyte alga *Hemiselmis andersenii *CCMP644 is presented.

**Results:**

The *H. andersenii *mtDNA is 60,553 bp in size and encodes 30 structural RNAs and 36 protein-coding genes, all located on the same strand. A prominent feature of the genome is the presence of a ~20 Kbp long intergenic region comprised of numerous tandem and dispersed repeat units of between 22–336 bp. Adjacent to these repeats are 27 copies of palindromic sequences predicted to form stable DNA stem-loop structures. One such stem-loop is located near a GC-rich and GC-poor region and may have a regulatory function in replication or transcription. The *H. andersenii *mtDNA shares a number of features in common with the genome of the cryptophyte *Rhodomonas salina*, including general architecture, gene content, and the presence of a large repeat region. However, the *H. andersenii *mtDNA is devoid of inverted repeats and introns, which are present in *R. salina*. Comparative analyses of the suite of tRNAs encoded in the two genomes reveal that the *H. andersenii *mtDNA has lost or converted its original *trnK(uuu) *gene and possesses a *trnS*-derived '*trnK(uuu)*', which appears unable to produce a functional tRNA. Mitochondrial protein coding gene phylogenies strongly support a variety of previously established eukaryotic groups, but fail to resolve the relationships among higher-order eukaryotic lineages.

**Conclusion:**

Comparison of the *H. andersenii *and *R. salina *mitochondrial genomes reveals a number of cryptophyte-specific genomic features, most notably the presence of a large repeat-rich intergenic region. However, unlike *R. salina*, the *H. andersenii *mtDNA does not possess introns and lacks a Lys-tRNA, which is presumably imported from the cytosol.

## Background

The mitochondrion is a double-membrane enclosed organelle found in the vast majority of extant eukaryotes. Mitochondria are best known for their essential role in energy generation, but they are also the site of additional important cellular processes such as iron-sulfur (Fe-S) cluster assembly and the beta-oxidation of fatty acids [1]. Some degenerate forms of mitochondria, such as the mitosome of the diplomonad parasite *Giardia lamblia*, have secondarily lost energy generating pathways and seem to retain only the Fe-S cluster maturation function [2]. All mitochondria are believed to share a single origin from an α-proteobacterial-like prokaryote [1], but a wide diversity of mitochondrial genome architectures have evolved subsequent to the diversification of modern-day eukaryotes [1,3,4]. For example, whereas "derived" animals possess monomeric circular mitochondrial genomes, an observation which led to the initial assumption that mtDNAs are primarily circular [5], many other mitochondrial genomes, such as that of the ciliate *Tetrahymena pyriformis *[6], the green alga *Chlamydomonas reinhardtii *[7] and the cnidarian metazoan *Aurelia aurita *(moon jelly) [8] are linear [9]. In addition, while some fungi and many plants have circular-mapping mtDNAs, their mitochondria actually contain predominantly linear mtDNA molecules with combinations of monomers and concatemers, with only a minor fraction of the molecules present in a circular form [10]. A more extreme example is the mtDNA of kinetoplastids, which consists of one maxi- and many different mini-circles that are interconnected to form an extensive network [11]. Mitochondrial gene content is also highly variable; the mtDNA of the jakobid flagellate *Reclinomonas americana *encodes 97 genes, the largest set of mitochondrial genes currently known [12], whereas the mtDNA of the malaria parasite *Plasmodium falciparum *contains just 3 protein coding genes and 2 highly fragmented small and large subunit ribosomal RNA (rRNA) genes [13]. The most highly derived forms of mitochondria, such as the hydrogenosome of *Trichomonas vaginalis *[14] and the *Giardia lamblia *mitosome [2], have lost their genomes entirely [15].

Mitochondria are also known as sites of unusual molecular biology and biochemistry. Marande and Burger [3] recently showed that the mtDNA genes of the euglenid *Diplonema papillatum *are fragmented into as many as nine modules, each residing on a distinct 6 or 7 Kbp chromosome. The mechanism by which these fragmented gene pieces are linked together to form contiguous transcripts is unknown. Extensive mRNA editing is another example of the bizarre molecular biology of mitochondria. Kinetoplastid mitochondrial mRNAs are subject to insertions and deletions of uridylate residues, sometimes >100 such insertions/deletions per transcript [16]. Mitochondrial mRNA editing is also widespread in land plants [17] and dinoflagellates [18]. For example, ~2% of the *cox1 *and *cob *gene sequences in three dinoflagellate species investigated by Lin et al. [19] were edited at the mRNA level.

We are studying the genomic diversity and evolution of cryptophytes, a ubiquitous and ecologically significant group of single-celled eukaryotes found in freshwater and marine environments. Most cryptophytes, except for members of the genus *Goniomonas*, harbor plastids of secondary endosymbiotic origin [20]. A variety of shared morphological features, such as the presence of ejectisomes, flat mitochondrial cristae, and an anterior depression, support the monophyly of cryptophytes, as do molecular phylogenetic data [21]. One unique feature of cryptophyte plastids that distinguishes them from other plastids of red algal origin is the retention of the remnant nucleus of the red algal endosymbiont, referred to as the nucleomorph [22,23]. Consequently, most cryptophytes harbor four distinct genomes–nuclear, nucleomorph, mitochondrial, and plastid genomes–contained in separate compartments. Cryptophytes are thus an interesting model system with which to study endosymbiotic gene transfer, genome evolution, and protein targeting.

In this study, we report the complete mitochondrial genome sequence of the newly described cryptophyte species *Hemiselmis andersenii *CCMP644, and compare it to the only other cryptophyte mitochondrial genome described thus far, that of *Rhodomonas salina *[24]. In addition, individual and concatenated mitochondrial protein coding gene sequences were analyzed to infer the phylogenetic relationships of cryptophytes to other eukaryotes.

## Methods

### DNA preparation, sequencing, and genome assembly

*Hemiselmis andersenii *mtDNA was isolated and sequenced to ~10× coverage as described in Lane et al. [25]. About 1,200 end sequences were screened for quality and vector contamination with Pregap4 and automatically assembled using gap4 version 4.10 in the Staden package [26]. Complete automated assembly of a large intergenic space between *trnS *and *cox2 *was unsuccessful due to the highly repetitive nature of this region. In an attempt to manually resolve this area, short (~30 bp) unique sequences within the *trnS *and *cox2 *genes were used to probe the sequence database for reads that extended from these two loci into the repeat region. These sequences were extracted and manually aligned using MacClade version 4.08 [27]. Sequences at the ends of the new constructs were then selected and the process was repeated. However, due to the presence of multiple identical copies of a >500 bp repeat, the assembly of a single unambiguous contig was not possible. When all available sequence reads were considered, three robust contigs were produced, each ending with similar repetitive sequences consisting of a ~340 bp repeat unit. These three contigs were joined to circularize the map. The complete *H. andersenii *mtDNA has been submitted to GenBank under the following accession number: EU651892.

DNA secondary structure within the repeat region was predicted using mfold version 3.2 [28] at a folding temperature of 37°C and the ionic conditions of 1.0 M [Na^+^] and 0.0 M [Mg^++^].

### Genome size/structure determination

We used pulsed-field gel electrophoresis (PFGE) to obtain an independent size estimate of the *H. andersenii *mitochondrial genome. *Hemiselmis andersenii *total DNA plugs were prepared as described in Lane *et al*. [29] and digested overnight with the restriction enzymes *Pst*I or *Bgl*II (Fermentas, Hanover, MD, USA). Based on the genome sequence, these enzymes were predicted to cut the mtDNA only once or twice. Both untreated and enzyme-digested *H. andersenii *DNA plugs were run on a 1% agarose gel (1× TBE) in 0.5× TBE buffer at 14.0°C for 18 h at a voltage of 6.0 V/cm with a switch time between 1–25 s using a CHEF-DR III Pulsed-Field Gel Electrophoresis System (Bio-Rad Laboratories, Hercules, CA, USA). DNA on the pulsed-field gel was transferred to a nylon membrane. Southern hybridization using a ~700 bp *cox*I probe as in Lane and Archibald [30] revealed that undigested mitochondrial DNA molecules were trapped in the wells or found in the 'compression zone'. The *Pst*I or *Bgl*II endonuclease treated DNA plugs revealed mitochondrial molecules in a discrete band below the 'compression zone'. The corresponding bands could not be visualized on the ethidium-bromide stained pulsed-field gel image because of nuclear and nucleomorph DNA smears in the background. In order to visualize mtDNA on the pulsed-field gel, an initial PFGE run was used to remove the linear nuclear and nucleomorph chromosomes from the PFGE plugs. These plugs, which still contained organellar DNA, were subsequently removed from the gel and digested with the restriction enzymes *Pst*I and *Bgl*II. Digested plugs were then inserted into a fresh gel and electrophoresed under the conditions described above. The 5 Kbp and Lambda CHEF DNA Size Standard (Bio-Rad Laboratories, Hercules, CA, USA) were used to estimate the size of the enzymatically linearized *H. andersenii *mtDNA.

### Genome annotation and GC content/skew analyses

Annotation of the *H. andersenii *mtDNA and the GC content and skew analyses were performed in Artemis version 8 [31]. Gene identification was carried out using BLASTX and BLASTN. Small and large ribosomal rRNA subunit genes were identified by comparison to rRNA gene sequences in the mitochondrial genome of *Rhodomonas salina*. Transfer RNAs were identified using tRNAscan-SE version 1.21 [32].

### Genome rearrangements between the two cryptophyte mtDNA

The extent to which the *H. andersenii *and *R. salina *mitochondrial genomes are rearranged to each other was estimated using GRIMM [33]. Each genome was designated as a sequence of 63 units, which include a repeat region and 62 genes common between the two cryptophyte mtDNAs.

### RT-PCR of '*trnK(uuu)*'

tRNAscan-SE version 1.21 [32] identified a putative intron of ~20 bp in the anticodon loop of the *H. andersenii trnK(uuu) *gene. To determine whether this prediction was correct, we performed RT-PCR using Lysine-tRNA-specific primer pairs and *H. andersenii *total RNA provided by H. Khan. To eliminate DNA contamination, 1 μl of total RNA was incubated for 30 min with RQ1 RNase-Free Dnase (Promega, Madison, WI, USA). RT-PCR was performed using the QIAGEN one-step RT-PCR kit (QIAGEN, Valencia, CA, USA) and with control reactions in which the reverse-transcription process was skipped. The following two pairs of primers were used: 1) The forward primer 5'-GAAGGTTGCTCGAATGGAA-3' with the reverse primer 5'-GAAGGTATAGGAATTGAACCTATTC-3' 2) and the forward primer 5'-GCCCAGAAGGTTGCTC-3' with the reverse primer 5'-AAGAAGGTATAGGAATTGAACCTAT-3'. RT-PCR was performed with the reverse transcription step for 30 min at 50°C and the subsequent inactivation of reverse transcriptase and activation of HotStart Taq DNA polymerase for 15 min at 95°C, followed by 35 cycles at 94°C for 1 min, 47°C for 1 min, and 72°C for 1 min, and a final extension at 72°C for 10 min. The amplified PCR fragments were cloned into pCR4-TOPO vector in the TOPO TA cloning kit for sequencing (Invitrogen, Carlsbad, CA, USA). Between 5 and 10 bacterial colonies from each reaction were selected for sequencing on a Beckman Coulter CEQ8000 (Beckman Coulter Inc., Fullerton, California, USA).

### Molecular phylogenetic analysis

From the 36 protein-coding genes found in the *H. andersenii *mtDNA, 25 were selected for phylogenetic analyses. Eleven genes (*atp8, nad8, rps2, rps3, rps4, rps7, rps8, rps13, rpl5, rpl6, tatC*) were excluded because their sequences were poorly conserved and/or were only present in a few taxonomic groups. *H. andersenii *protein sequences were aligned with their homologs from other mitochondrial genomes available from GenBank. Amino acid sequences were aligned using MacClade version 4.08 [27] and ambiguously aligned sites were manually removed. In addition to individual protein analyses, a concatenated protein data set containing 25 proteins was analyzed. To include the maximum number of gene sequences, we combined 25 protein-coding gene sequences encoded in 18 mitochondrial genomes across diverse eukaryotic taxa. As most mitochondrial genomes do not possess all 25 protein-coding genes selected for analysis, as many as 12 protein gene sequences were missing per taxon. A maximum likelihood tree was produced using RAxML-VI-HPC version 2.2.3 [34] with the PROTOMIXJTT model of sequence evolution and the automatic tree rearrangement setting, and from 100 distinct randomized maximum parsimony starting trees. Bootstrap analysis was based on 100 re-samplings.

## Results and Discussion

### General features of *Hemiselmis andersenii *mtDNA

**Figure 1:**
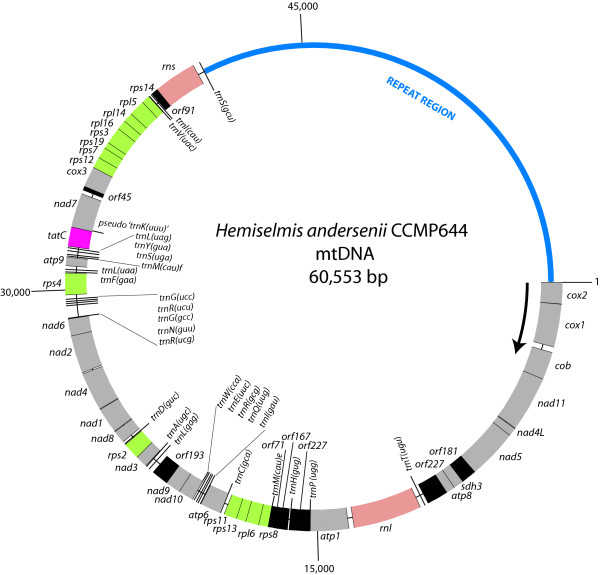
**Circular map of the mitochondrial genome of the cryptophyte *Hemiselmis andersenii***. All of the genes are transcribed in a clockwise direction. Note the dense gene arrangement and a single large intergenic region. Protein-coding genes and ribosomal RNA genes are labeled outside the circle, whereas transfer RNAs and open reading frames of unknown functions are labeled on the inside. '*TrnK(uuu)*' may be a pseudogene (see main text for discussion). Genes are color-coded according to functional categories: green for ribosomal protein genes, gray for genes involved in oxidative phosphorylation, pink for the protein translocase protein gene *tatC*, salmon for ribosomal subunit genes, and black for open reading frames with unknown functions.

The mitochondrial DNA of the cryptophyte *Hemiselmis andersenii *CCMP644 was sequenced, assembled and manually edited to produce a circular-mapping genome 60,553 bp in size (Figure 1). Genome assembly was complicated by the presence of a highly repetitive non-coding region of ~20 Kbp (see below); genome size was thus verified using pulsed-field gel electrophoresis (PFGE). Several observations suggest that the *H. andersenii *mtDNA exists primarily in a linear-branched form comprised of multiple genome units. In PFGE, the *H. andersenii *mtDNA remains in the well or migrates within the 'compression zone' (i.e., the unresolved portion of DNA near the top of the gel), which contains primarily linear nuclear and nucleomorph chromosomes larger than ~150 Kbp (data not shown). The lack of mtDNA below the 'compression zone' suggests that the *H. andersenii *mtDNA is not composed of linear monomers or dimers. Furthermore, when the *H. andersenii *mtDNA is partially digested with *Pst*I, an enzyme predicted to cut the genome only once, it produces a discrete band of ~60 Kbp in size (data not shown) but not a band ~120 Kbp in size, which would correspond to a dimeric linear form of the genome. This result indicates that the *H. andersenii *mtDNA is not composed of circular concatemers or linear head-to-tail concatemers consisting of three or more genomic units. Therefore, we suggest that the *H. andersenii *mtDNA exists primarily as a branched linear molecule although monomeric circles may also exist. Further studies using transmission electron microscopy or the 'moving picture' technique [10] will be necessary to confirm this hypothesis.

**Figure 2:**
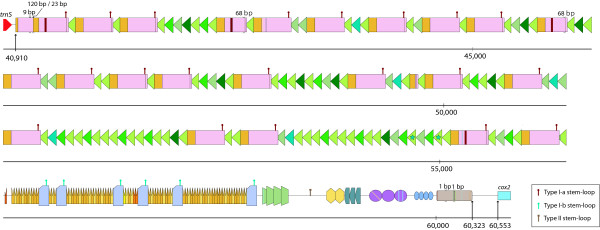
**Schematic diagram of the large repeat region in the *Hemiselmis andersenii *mitochondrial genome**. This region is ~20 Kb in size and includes multiple repeat units arranged in tandem or dispersed among tandem repeats. Slight variations of each repeat unit are color-coded and/or marked with strips or a star symbol. Predicted nucleotide deletions within a repeat unit are highlighted with arrowheads: the size of the deletion is also provided. The positions of three kinds of DNA stem-loop forming sequences, Type I-a, I-b, and II, are labeled with "hairpin" symbols.

**Figure 3:**
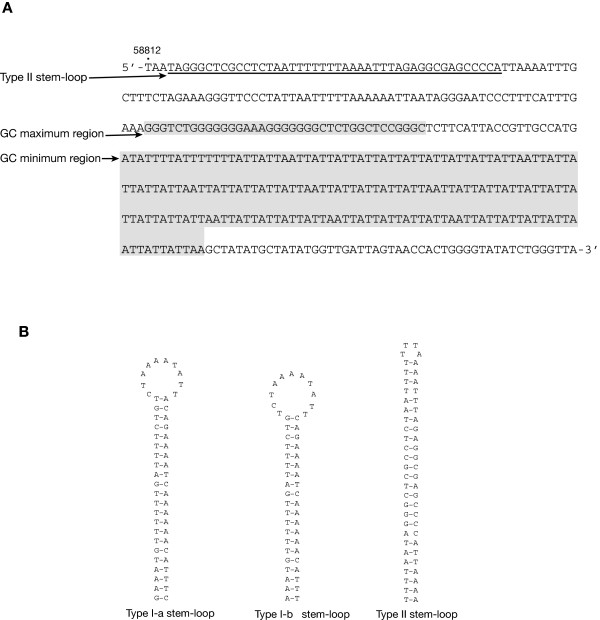
**(A) Isolated region of the *Hemiselmis andersenii *mitochondrial genome located near the 3' end of the large intergenic space.** This area includes a palindromic sequence that is predicted to form a Type II stem-loop followed by high and low GC regions. A similar region is not found in the *R. salina *mtDNA. **(B) Three predicted stem-loop structures found within the large intergenic space.** Note that Type I-a and I-b differ only by 3 nucleotides.

The *H. andersenii *mitochondrial genome is comprised of a gene-rich region ~40 Kbp in size and a large (19,675 bp) intergenic region between *trnS *and *cox2 *with complex repeats (Figures 1 and 2). The intergenic region accounts for 32.5% of the entire genome and 83.5% of the total amount of non-coding DNA (23,549 bp). The overall GC content of the genome is 28.72%, slightly higher than that of the nucleomorph genome of this organism [25]. Interestingly, a ~40 bp region near the start of the coding portion of the genome is very GC-rich (78.38%) and is followed by a 100% AT-containing region ~190 bp in size (Figure 3). This unusual stretch of sequence is about 70 bp from a palindromic sequence that is predicted to form a Type II stem-loop (Figures 2 and 3; see discussion below), and may be involved in regulating replication or transcription.

The *H. andersenii *mitochondrial genome encodes 66 genes with predicted functions and 8 hypothetical protein-coding genes, a total somewhat higher than the average for eukaryotes (40–50 genes) [1]. Ten genes–*orf167, orf71, rps13, rps11, nad3, rps2, tatC, 'trnK (uuu)', rps12*, and *rps7*–overlap by up to 51 bp, emphasizing the extreme compactness of the coding portion of the genome. The genome encodes small and large rRNA subunit genes and 28 tRNAs, one of which may be a pseudogene (see discussion below). Of the 36 identifiable protein-coding genes, 14 encode ribosomal proteins, 21 are involved in oxidative phosphorylation, and one gene encodes a membrane translocase protein (Table 1).

**Table 1:** Functional categories of 36 protein genes encoded in the mitochondrial genome of *Hemiselmis andersenii*.

Protein categories	Sub-categories	Genes
Ribosomal proteins (14)	Small subunit	*rps2 rps3 rps4 rps7 rps8 rps11 rps12 rps13 rps14 rps19*
	Large subunit	*rpl5 rpl6 rpl14 rpl16*
Oxidative phosphorylation (21)	NADH dehydrogenase	*nad1 nad2 nad3 nad4 nad4L nad5 nad6 nad7 nad8 nad9 nad10 nad11*
	Ubiquinol:cytochrome c oxidoreductase	*cob*
	Succinate:ubiquinone oxidoreductase	*sdh3*
	Cytochrome c oxidase	*cox1 cox2 cox3*
	ATP synthase	*atp1 atp6 atp8 atp9*
Sec-independent protein translocase protein (1)		*tatC*

Comparison of the mtDNA gene order in *H. andersenii *to other genomes reveals the presence of five gene clusters shared among distantly related protists: two ribosomal protein clusters (*rps12-rps7-rps19-rps3-rpl16-rpl14-rpl5-rps14 *and *rps8-rpl6-rps13-rps11*) and three NADH dehydrogenase clusters (*nad4L-nad5*; *nad4-nad2*; *nad10-nad9*). These gene clusters have been suggested to represent vestiges of bacterial operons [12,24]. Interestingly, all 74 genes in the *H. andersenii *mitochondrial genome are encoded on the same strand. While the evolution of such an arrangement seems improbable, absolute strand polarity has been observed in the mitochondrial genomes of diverse eukaryotes such as the amoeba *Acanthamoeba castellanii *(59 genes), the fungus *Penicillium marneffei *(47 genes), and the green alga *Chlamydomonas eugametos *(20 genes) [35-37]. In addition, strikingly similar mtDNA architectures–gene-dense regions, a single large repetitive intergenic region, and all genes encoded on one strand–are seen in diverse protists such as the stramenopile *Thraustochytrium aureum *(The Organelle Genome Megasequencing Program; http://megasun.bch.umontreal.ca/ogmp/) and the green alga *Pedinomonas minor *[38]. Understanding the biological significance of such convergence at the level of genome architecture will require comparative molecular and biochemical studies of mitochondria in these organisms.

### Comparison of the mitochondrial genomes of *Hemiselmis andersenii *and *Rhodomonas salina*

*H. andersenii *is only the second cryptophyte, after *R. salina *[24], for which a mitochondrial genome has been completely sequenced and annotated. Comparative analyses of the two genomes revealed a number of similarities. Both genomes feature a compact gene arrangement and a single large repeat region (Figure 1) [24], although the size of the large intergenic region in *H. andersenii *(~20 Kbp) is more than four times as large as that of *R. salina *(~4.7 Kbp). All of the 36 predicted protein-coding genes in the *H. andersenii *mitochondrial genome are present in the *R. salina *mtDNA. Four *R. salina *mitochondrion-encoded genes–*rps1, atp4, tatA*, and *sdh4*–are not found in *H. andersenii*, although two open reading frames, *orf45 *and *orf91*, in the *H. andersenii *mtDNA show marginal sequence similarity to the *R. salina tatA *and *sdh4 *genes, respectively. Additionally, while two group II introns are present in *R. salina *mtDNA, the *H. andersenii *mtDNA is devoid of introns (Table 2) [24].

**Table 2:** Comparison of two cryptophyte mitochondrial genomes

	*Hemiselmis andersenii*	*Rhodomonas salina*
Genome size	60, 553 bp	48,063 bp
Coding capacity	61%	69%
GC %	29%	29%
Size of the repeat region	19.7 Kbp (33%)	4.7 Kbp (10%)
Group II introns	not present	two
Number of genes (with assignable functions)	66 genes (28 tRNAs)	69 genes (27 tRNAs)
Inverted repeats	not present	a pair of ~1.5 Kbp repeat units

With respect to conservation of gene order, 64.5% of the shared genes between the two cryptophyte mitochondrial genomes (40 out of 62 genes–36 protein-coding genes, 24 tRNA genes (see below), 2 rRNA genes) are present in thirteen syntenic blocks, each consisting of 2–7 genes. These include: 1) *cox1-cob-nad11*, 2) *nad4L-nad5*, 3) *atp1-trnP(ugg)*, 4) *rps8-rpl6-rps13-rps11*, 5) *trnC(gca)-atp6*, 6) *trnI(gau)-trnQ(uug)-trnR(gcg)-trnE(uuc)-trnW(cca)-nad10-nad9*, 7) *nad4-nad2*, 8) *trnR(ucu)-trnG(ucc)*, 9) *trnM(cau)f-trnS(uga)*, 10) *trnY(gua)-trnL(uag)*, 11) *tatC-'trnK(uuu)' *[*H. andersenii*] */trnS(gcu) *[*R. salina*]-*nad7*, 12)*cox3-rps12-rps7-rps19*, and 13) *rps3-rpl16-rpl14-rpl5-rps14*. As noted earlier, some of the conserved gene clusters, such as *nad4L-nad5*, are found in distantly related eukaryotes and appear to be vestiges of bacterial operons. Analysis using GRIMM [33] suggests that the observed difference in gene order between the two cryptophyte mitochondrial genomes can be explained by at least 31 instances of genome reversal events.

### Repeat structure of the *H. andersenii *mitochondrial genome

The *R. salina *mtDNA is characterized by a pair of ~1.5 Kbp inverted repeats that are joined by 112 bp of sequence [24]. In contrast, repeats in the *H. andersenii *mitochondrial genome are not inverted, but are instead dispersed or arranged in tandem throughout the large non-coding region, with individual repeat units ranging from 22 to 336 bp and occurring up to 100 times (Figure 2). Given that *R. salina *and *H. andersenii *are distantly related to one another [29], the large repeat region presumably arose during or prior to the early diversification of cryptophytes. While there is no obvious sequence similarity between the two repeat regions, both contain multiple copies of palindromic sequences, which are predicted to form stable stem-loop DNA structures [24]. In *H. andersenii*, two types of stem-loop structures were identified–I and II–using the DNA MFOLD program [28]. The Type I structure has two slight variations, I-a and I-b, which occur 21 and 5 times, respectively (Figures 2 and 3). Type I-a and I-b structures have 22 and 20 base pairings in their stems, respectively, and occur adjacent to tandem repeats (Figures 2 and 3). One copy of the type II stem-loop structure is located within a ~300 bp segment that is devoid of any discernable repeat units, but close to the high and low GC regions noted earlier (Figures 2 and 3). As was suggested for *R. salina *by Hauth et al. [24], tandem repeats and multiple stem-loop structures in *H. andersenii *mtDNA might be involved in the regulation of transcription and replication, a hypothesis that needs to be tested further.

Hauth et al. [24] demonstrated that the repeat region of the *R. salina *mtDNA roughly coincides with a change in the direction of 'cumulative GC skew' [calculated as (G-C)/(G+C)] and suggested that the repeat corresponds to the origin of replication. We investigated the GC skew in the *H. andersenii *mitochondrial genome to see whether a similar pattern exists. Unlike *R. salina*, however, the *H. andersenii *GC skew does not change direction near the repeat region. Instead, in both the *H. andersenii *and *R. salina *mtDNA, observed GC skew patterns strongly correlate with transcriptional orientations, where the coding strand tends to be G-rich (data not shown). Therefore, the GC skew patterns of the two cryptophyte mitochondrial genomes do not seem to be the result of replication-associated mutational bias, but rather the non-random distribution of the protein coding genes, as has been observed in some other genomes [39]. Nevertheless, based on the presence of other features such as stem-loop structures, it seems reasonable to assume that the repeat region in both cryptophyte mitochondrial genomes corresponds to the origin of replication.

### Codon usage and transfer RNAs

**Figure 4:**
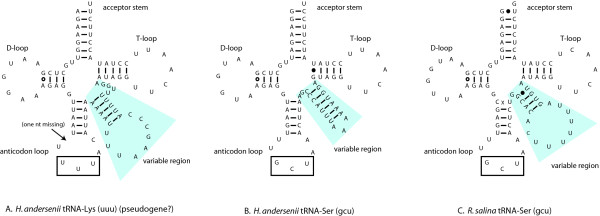
**Predicted secondary structures for three homologous cryptophyte tRNAs.***Hemiselmis andersenii *'*trnK(uuu)' *(A) is paralogous to *trnS(gcu) *(B). Duplication and divergence of an ancestral *trnS(gcu) *appears to have led to the evolution of '*trnK(uuu)*' in *H. andersenii *(A), which consequently, possesses an atypically long variable region. The anticodon loop and the variable region of '*trnK(uuu)*' is AT-rich and the predicted stem regions consist entirely of A-T base pairings. Furthermore, the loop of the anticodon loop/stem region is missing one nucleotide (arrow). These structural considerations suggest that '*trnK(uuu)*' may not be functional. The '*trnK(uuu)' *gene in *H. andersenii *(A) is orthologous to *trnS(gcu) *in *R. salina *(C). Note that while sequences within the anticodon loop, V-arm, and acceptor stem are divergent between the two orthologous copies, sequences within the D loop and the T loop are conserved.

The *H. andersenii *mtDNA encodes 28 tRNAs, 27 of which are predicted to form standard cloverleaf secondary structures. One tRNA gene, '*trnK(uuu)*', shows atypical structure in the anticodon loop and the variable region, and is probably a pseudogene (Figure 4A). Allowing for wobble pairings and some base modifications, 26 tRNAs are the theoretical minimum required to cover all codons in bacteria. For some mitochondria, even smaller sets of tRNAs, as few as 22–23, are possible by adopting several additional strategies [40]. The *H. andersenii *mitochondrial genome lacks only one tRNA gene, *trnK(uuu)*, which is minimally required in order to recognize all 61 codons (Table 3). It is thus predicted that nuclear-encoded cytosolic Lys-tRNA is imported into *H. andersenii *mitochondria. Mitochondrial tRNA import has been demonstrated in apicomplexans and trypanosomatids where tRNA genes are completely missing in their mitochondrial genomes [41], as well as in ciliates and plants where mitochondrial genomes encode fewer than the 22–23 minimally required tRNA genes [42]. Although most animals and some fungi do not import tRNAs into mitochondria [43], the fungus *Saccharomyces cerevisiae *has been shown to import one specific cytosolic tRNA even though its mitochondrial genome encodes the full complement of tRNAs [44]. Analyses of the tRNA repertoire of mitochondrial genomes suggest that a number of other protist taxa across the eukaryotic tree also import one or more tRNAs into their mitochondria [43,45]. It is thus reasonable to assume that *H. andersenii *imports at least Lys-tRNA, although it is possible that tRNA editing makes up for the Lys-tRNA deficit by changing the identity of an existing tRNA, as has been shown in marsupials [46].

**Table 3:** *Hemiselmis andersenii *mtDNA codon usage table.

		Second Position of Codon					
		
		U	C	A	G		
		
First Position of Codon	U	UUU [F] 608	UCU [S] 123	UAU [Y] 312	UGU [C] 123	U	Third Position of Codon
		UUC [F] 126•	UCC [S] 18	UAC [Y] 69•	UGC [C] 20•	C	
		UUA [L] 688•	UCA [S] 205•	UAA [stop] 32	UGA [stop] 2	A	
		UUG [L] 163	UCG [S] 57	UAG [stop] 3	UGG [W] 120•	G	
		
	C	CUU [L] 179	CCU [P] 139	CAU [H] 140	CGU [R] 83	U	
		CUC [L] 38•	CCC [P] 18	CAC [H] 30•	CGC [R] 17•	C	
		CUA [L] 88•	CCA [P] 115•	CAA [Q] 217•	CGA [R] 95•	A	
		CUG [L] 20	CCG [P] 28	CAG [Q] 42	CGG [R] 32	G	
		
	A	AUU [I] 579	ACU [T] 162	AAU [N] 307	AGU [S] 183	U	
		AUC [I] 85•	ACC [T] 24	AAC [N] 95•	AGC [S] 29•	C	
		AUA [I] 207•	ACA [T] 242•	AAA [K] 517• †	AGA [R] 110•	A	
		AUG [M] 233••	ACG [T] 54	AAG [K] 75	AGG [R] 15	G	
		
	G	GUU [V] 311	GCU [A] 186	GAU [D] 221	GGU [G] 299	U	
		GUC [V] 38	GCC [A] 35	GAC [D] 39•	GGC [G] 41•	C	
		GUA [V] 204•	GCA [A] 225•	GAA [E] 283•	GGA [G] 131•	A	
		GUG [V] 62	GCG [A] 64	GAG [E] 65	GGG [G] 83	G	

The frequency of each codon is shown and dots indicate the presence of a corresponding tRNA gene in the mtDNA. †: '*trnK(uuu)*' identified in the genome may be a pseudogene (see discussion).

Another possible mechanism to account for the missing tRNA is that the structurally abnormal '*trnK(uuu)*' gene (Figure 4A) forms a functional Lys-tRNA to decode the codons AAA and AAG. Several cases of atypically-structured tRNAs are known from animal and ciliate mitochondria [47,48]. Interestingly, tRNAscan-SE [32] predicted the existence of a 20 bp intron within the *H. andersenii *'*trnK(uuu)'*, and we conducted further experiments to test whether this is indeed the case. RT-PCR experiments using primer sets specific for '*trnK(uuu)*' indicated that the putative intron was not removed in the mature tRNA. This results is not unexpected, given that the 20-bp putative intron is too short to be a self-splicing group I or II intron, which are the only known types of introns reported in mitochondrial genomes [49]. Sequencing of ~20 clones also did not reveal any evidence for RNA editing within the '*trnK(uuu)*'. These results suggest that if '*trnK(uuu)*' is indeed expressed to form a functional Lys-tRNA, it is predicted to have an unusually AU-rich stem in the codon loop and a long variable region, atypical for Lys-tRNA (Figure 4A). Long variable regions ranging from 11 to 23 nucleotides are generally restricted to tRNA-Leu, tRNA-Ser, and bacterial tRNA-Tyr [40]. The D- and T-loops of the '*trnK(uuu)*' sequence show sequence similarity to one of the two mitochondrion-encoded tRNA-Ser genes (Figure 4A and 4B), both of which have a long variable region. In addition, comparative analysis with the *R. salina *mtDNA revealed genomic position conservation between the *H. andersenii trnS*-like *'trnK(uuu)' *gene and the *trnS(gcu) *gene of *R. salina*, flanked by the *tatC *and *nad7 *genes. The *H. andersenii *'*trnK(uuu)*' and *R. salina trnS(gcu) *genes both overlap *tatC *by 51 bp and 22 bp, respectively. This strongly suggests that the *H. andersenii 'trnK(uuu)' *is indeed derived from an ancestral gene that encoded tRNA-Ser, explaining the origin of its long variable region. The overlap between the *H. andersenii *'*trnK(uuu)*' and *tatC *suggests that '*trnK(uuu)*' may play a role in processing the 3' end of the *tatC *gene transcript. This hypothesis could explain why the '*trnK(uuu)*' gene still remains in the genome and retains conserved secondary structure in the stem loop and D- and T-loops, even if it does not form a functional tRNA. Comprehensive molecular and biochemical experimentation will be necessary to confirm or refute the existence of mitochondrial tRNA import in *H. andersenii *and the functionality of the unusual '*trnK(uuu)*' gene.

When the *H. andersenii *tRNA genes were compared to those of *R. salina*, 24 homologous pairs of tRNAs were identified, leaving only four *H. andersenii *tRNA and three *R. salina *tRNA genes not unambiguously matched to each other. Each of the tRNA pairs possess identical anticodons except for the *H. andersenii 'trnK(uuu)' *and *R. salina trnS(gcu) *pair, despite their common derivation. The *trnS(gcu) *of *H. andersenii*, having sequence homology to the '*trnK(uuu)*', probably originated from a recent gene duplication event. Of the three remaining *H. andersenii *tRNA genes that are unmatched in *R. salina*, two–*trnL(gag) *and *trnG(gcc)*–are redundant because *trnL(uag) *and *trnG(ucc) *can decode all of their respective four-codon families [40]. These redundant copies might have been lost in an ancestor of *R. salina *after it diverged from *H. andersenii*. Lastly, the *H. andersenii trnI(cau) *is somewhat similar to the *trnK(uuu) *of the *R. salina *and only marginally resembles the *R. salina trnI(cau) *at the 3' end. It is possible that the *H. andersenii trnI(cau) *originated through recombination between ancestral *trnI(cau) *and *trnK(uuu) *genes, which would explain the lack of an obvious *trnK(uuu) *homolog in *H. andersenii *comparable to the *R. salina trnK(uuu)*. Substantial sequence divergence among the three genes, however, makes it difficult to accurately trace the origin of the *trnI(cau) *and the loss of the original *trnK(uuu) *gene in *H. andersenii*. On the other hand, the unusual *trnI(uau) *gene reported from *R. salina *is not found in *H. andersenii*. It was suggested that the *R. salina trnI(uau) *is derived from *trnF(uuc) *through a recent gene duplication event [24]. Overall, the two cryptophyte mitochondrial genomes use similar tRNA sets to recognize codons. However, unlike *H. andersenii*, which may need to import at least *trnK(uuu) *from cytosol, the *R. salina *mtDNA does possess the minimal required set for tRNA autonomy.

### Molecular phylogenetic analyses

Cryptophytes are a well-established eukaryotic lineage, supported by both molecular and morphological features [20]. However, their relationship to other eukaryotic groups, particularly those containing plastids of secondary endosymbiotic origin, has been the subject of considerable debate. The cryptophyte plastid is the product of a secondary endosymbiosis involving a red algal cell, the same process which accounts for plastid origins in haptophytes, dinoflagellates, and stramenopiles [50]. Cavalier-Smith [50] suggested that plastids in these four algal lineages arose from a single secondary endosymbiosis in a common ancestor that these organisms shared, to the exclusion of other eukaryotic groups. However, this "chromalveolate" hypothesis is controversial [51,52]. Recent molecular studies have shown that the katablepharids, an enigmatic collection of plastid-less flagellates, are a sister group to cryptophytes [53,54], and large-scale concatenated analyses of nuclear genes suggest that cryptophytes and haptophytes are also related [55,56].

**Figure 5:**
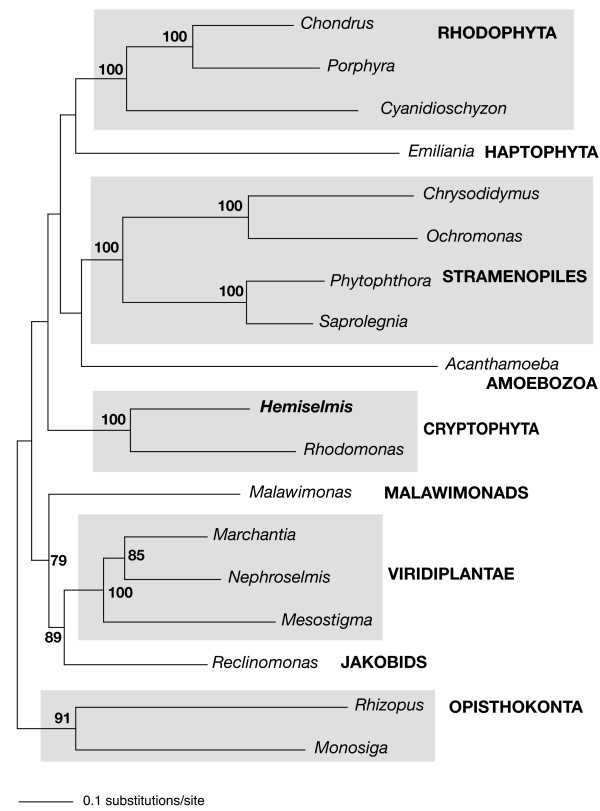
**Maximum likelihood phylogenetic tree based on 25 concatenated mitochondrial protein sequences inferred using RAxML.** Approximately 15% of data are missing. Bootstrap support values over 50% are indicated at the corresponding nodes.

To gain insight into the phylogenetic relationship of the cryptophytes *H. andersenii *and *R. salina *to other eukaryotes, and more specifically, to test the hypothesis that cryptophytes and haptophytes are related to one another, phylogenetic analyses of mitochondrial protein sequences were performed (Figure 5). Unlike the cryptophyte plastid genome, in which several cases of LGT have recently been discovered [57,58], individual analyses of 25 mitochondrial proteins did not reveal any obvious instances of LGT between prokaryotes and eukaryotes or within eukaryotes (data not shown). However, the possibility of ancient LGTs cannot be ruled out, as the backbones of individual protein phylogenies were generally very poorly supported.

As expected, a close relationship between the two cryptophytes *H. andersenii *and *R. salina *was well supported in the mitochondrial protein phylogenies, with twenty of twenty-five individual protein phylogenies showing this relationship. Five individual gene phylogenies–*nad2, rpl14, rpl16, rps12, rps14*–did not recover a *H. andersenii*-*R. salina *clade, although alternative topologies were not supported with >50% bootstrap support values. Additionally, single protein phylogenies were not, for the most part, able to resolve the relationship of cryptophytes to other eukaryotes. The position of cryptophytes was highly variable from protein to protein and the group did not regularly associate with other taxonomic clades with >50% bootstrap support values, except for in the *cob *and *nad1 *gene trees, where cryptophytes branch with haptophytes (81%) and jakobids (77%), respectively.

We subsequently analyzed a set of 25 concatenated proteins to assess the phylogenetic position of cryptophytes. In this analysis, the *H. andersenii*-*R. salina *clade received 100% bootstrap support (Figure 5). Other well-established eukaryotic groups including opisthokonts, rhodophytes, stramenopiles, and Viridiplantae, were also strongly recovered, but the relationships among major lineages were not. The jakobid *Reclinomonas *branched as the sister group to the Viridiplantae with moderate support (89% bootstrap support), and *Malawimonas *showed an affinity for these two groups in two of the three data sets, as was previously inferred from a concatenate of ten mitochondrial proteins [59]. It is not clear whether the jabokid (and/or malawimonad)-Viridiplantae affinity is a phylogenetic artifact or reflects the true evolutionary history of mitochondrial genes. Though growing evidence supports a relationship between cryptophytes and haptophytes [55,56,58], our extensive mitochondrial protein analyses did not reveal this relationship with reasonable bootstrap support, other than in a single protein gene tree (*cob)*. In summary, while mitochondrial gene sequences are able to resolve some of the eukaryotic lineages determined using other markers, they are at present incapable of resolve the deepest branches of the eukaryotic tree using current phylogenetic methods and with the present level of taxon sampling.

## Conclusion

We have sequenced the mitochondrial genome of the cryptophyte *H. andersenii *and compared it to that of the distantly related cryptophyte *R. salina*. Our analyses reveal that both genomes are characterized by a gene dense region and a single large intergenic space that includes numerous repeats and palindromic sequences predicted to form stable DNA stem and loop structures. Despite the overall similarities in content and architecture between the two genomes, their modes of regulating DNA replication and transcription seem to differ. Unlike *R. salina*, all 73 genes in the *H. andersenii *mtDNA are located on the same strand, a relatively rare observation in mitochondrial genomes. Phylogenic analysis of multiple mitochondrial gene sequences indicated a clear affiliation between the two cryptophytes but was not able to resolve the position of cryptophytes relative to other eukaryotic groups.

## Authors' contributions

EK participated in genome assembly, carried out genome analysis and drafted the manuscript. CEL isolated *H. andersenii *DNA and participated in the initial genome assembly. BAC, CK, and SB performed the *H. andersenii *mitochondrial genome sequencing. JMA coordinated the study and helped draft the manuscript. All authors read and approved the manuscript.
